# From LI-RADS Classification to HCC Pathology: A Retrospective Single-Institution Analysis of Clinico-Pathological Features Affecting Oncological Outcomes after Curative Surgery

**DOI:** 10.3390/diagnostics12010160

**Published:** 2022-01-10

**Authors:** Leonardo Centonze, Riccardo De Carlis, Ivan Vella, Luca Carbonaro, Niccolò Incarbone, Livia Palmieri, Cristiano Sgrazzutti, Alberto Ficarelli, Maria Grazia Valsecchi, Umberto Dello Iacono, Andrea Lauterio, Davide Bernasconi, Angelo Vanzulli, Luciano De Carlis

**Affiliations:** 1 Department of General Surgery and Transplantation, Niguarda Ca’ Granda Hospital, 20161 Milan, Italy; riccardo.decarlis@ospedaleniguarda.it (R.D.C.); ivan.vella@ospedaleniguarda.it (I.V.); niccolo.incarbone@ospedalenigaurda.it (N.I.); livia.palmieri@ospedaleniguarda.it (L.P.); alberto.ficarelli@ospedaleniguarda.it (A.F.); andrea.lauterio@ospedaleniguarda.it (A.L.); luciano.decarlis@ospedaleniguarda.it (L.D.C.); 2 Department of Diagnostic and Interventional Radiology, Niguarda Ca’ Granda Hospital, 20161 Milan, Italy; luca.carbonaro@ospedaleniguarda.it (L.C.); cristiano.sgrazzutti@ospedaleniguarda.it (C.S.); umberto.delloiacono@ospedaleniguarda.it (U.D.I.); angelo.vanzulli@ospedaleniguarda.it (A.V.); 3 Bicocca Bioinformatics Biostatistics and Bioimaging Centre—B4, School of Medicine and Surgery, University of Milan-Bicocca, 20126 Milan, Italy; grazia.valsecchi@unimib.it (M.G.V.); davide.bernasconi@unimib.it (D.B.); 4 School of Medicine and Surgery, University of Milan-Bicocca, 20126 Milan, Italy

**Keywords:** HCC, Li-RADS classification, surgical resection, HCC recurrence, overall survival, cancer-specific survival, radiomics

## Abstract

*Background*: The latest Liver Imaging Reporting and Data System (LI-RADS) classification by the American College of Radiology has been recently endorsed in the American Association for the Study of Liver Disease (AASLD) guidelines for Hepatocellular carcinoma (HCC) management. Although the LI-RADS protocol has been developed as a diagnostic algorithm, there is some evidence concerning a possible correlation between different LI-RADS classes and specific pathological features of HCC. We aimed to investigate such radiological/pathological correlation and the possible prognostic implication of LI-RADS on a retrospective cohort of HCC patients undergoing surgical resection. *Methods*: We performed a retrospective analysis of the pathological characteristics of resected HCC, exploring their distribution among different LI-RADS classes and analyzing the risk factors for recurrence-free, overall and cancer-specific survival *Results*: LI-RADS-5 (LR-5) nodules showed a higher prevalence of microvascular invasion (MVI), satellitosis and capsule infiltration, as well as higher median values of alpha-fetoprotein (αFP) compared to LI-RADS-3/4 (LR-3/4) nodules. MVI, αFP, satellitosis and margin-positive (R1) resection resulted as independent risk factors for recurrence-free survival, while LI-RADS class did not exert any significant impact. Focusing on overall survival, we identified patient age, Eastern Cooperative Oncology Group performance status (ECOG-PS), Model for End Stage Liver Disease (MELD) score, αFP, MVI, satellitosis and R1 resection as independent risk factors for survival, without any impact of LI-RADS classification. Last, MELD score, log10αFP, satellitosis and R1 resection resulted as independent risk factors for cancer-specific survival, while LI-RADS class did not exert any significant impact. *Conclusions*: Our results suggest an association of LR-5 class with unfavorable pathological characteristics of resected HCC; tumor histology and underlying patient characteristics such as age, ECOG-PS and liver disease severity exert a significant impact on postoperative oncological outcomes.

## 1. Introduction

Hepatocellular carcinoma (HCC) incidence is constantly rising worldwide, representing the third leading cause of cancer-related death and 85–90% of primary liver neoplasms [1,2].

Following the European Association for the Study of the Liver (EASL) and the American Association for the Study of Liver Disease (AASLD) guidelines, HCC diagnosis relies on triphasic contrast enhanced imaging [3,4]. Back in 2011, the American College of Radiology proposed a standardized diagnostic algorithm for liver imaging reporting, named Liver Imaging Reporting and Data System (LI-RADS), that was furtherly updated in 2013, 2014 and 2017, until the release of the latest version in 2018 [5].

The 2018 LI-RADS protocol provides a 38% HCC-specific diagnostic accuracy for LI-RADS-3 (LR-3) observations, which raises up to 95% in LI-RADS-5 (LR-5) nodules [5,6]. Considering these different probabilities for harboring a HCC, current AASLD guidelines suggest early restaging or biopsy for LI-RADS-3/4 (LR-3/4) observations, recommending therapeutic approaches to LR-5 nodules [4].

Until this recent integration of LI-RADS protocol into clinical guidelines, HCC management was not supported by such probabilistic diagnostic tool: as a consequence, several LR-3/4 nodules underwent curative treatments such as percutaneous ablation (PA), surgical resection (SR) or liver transplantation (LT) [7], providing interesting subgroups for retrospective analysis.

Given this scenario, recent evidence supported a potential role of LI-RADS protocol on clinical decision making in the setting of HCC ablation vs. resection [8] and liver transplantation [9,10], suggesting an association of LI-RADS classes with specific pathological features of HCC nodules.

The aim of our study was to retrospectively assess the association of LR-3/4 and LR-5 subclasses with pathological characteristics of HCC treated by SR, investigating the risk factors for disease-free, overall and cancer-specific survival.

## 2. Materials and Methods

Study protocol followed the 1975 Declaration of Helsinki ethical guidelines, as revised in Brazil in 2013; all the subjects involved in the study gave their explicit informed consent for data collection and publication. Local ethical committees’ review of the protocol deemed that formal approval was not required owing to the retrospective, observational and anonymous nature of this study.

Results are reported according to Strengthening the Reporting of Observational Studies in Epidemiology (STROBE) [11].

### 2.1. Study Design

The study enrolled all adult (age ≥ 18 years) HCC patients undergoing SR between 2006 and 2016, with a retrievable pre-treatment contrast-enhanced imaging study.

The most recent preoperative imaging (either computed tomography or magnetic resonance imaging) was retrospectively and blindly analyzed by three radiologists with 30-, 15- and 10-year experience in liver imaging, classifying all the nodules according to 2018 Li-RADS protocol, applying ancillary features when feasible.

### 2.2. Data Collection and Definition

All data were retrieved from a single university-affiliated, hepato-pancreato-biliary teaching center prospective database and anonymized prior to analysis.

Baseline characteristics included patient age [years], gender, Eastern Cooperative Oncology Group performance status (ECOG-PS) [12], aetiology of liver disease, Child-Pugh [13], Albumin-Bilirubin (ALBI) [14] and Model for End Stage Liver Disease (MELD) [15] scores.

Tumor characteristics such as number and size [mm] of HCC nodules, as well as alpha-fetoprotein (αFP) value [ng/m], Barcelona Clinic for Liver Cancer (BCLC) staging [7] and LI-RADS class [5] were also collected.

Pathological analysis included the definition of tumor grading according to Edmondson–Steiner’s classification system [16], microvascular invasion (MVI) [17], satellitosis [18] and capsule infiltration [19], as well as the state of resection margins—margin-positive (R1) resection was defined as a <1 mm tumor distance from the cut surface.

Length of hospital stay [days] was collected, and postoperative complications were classified according to Clavien–Dindo classification [20].

The number of patients undergoing LT was also included.

Last, the date of tumor recurrence and date and cause of patient death were recorded during follow-up.

### 2.3. Perioperative Management and Surgical Technique

Every case was evaluated during multidisciplinary team (MDT) meetings involving surgeons, hepatologists, diagnostic and interventional radiologists. Notably, these MDT meetings were attended before the blinded reclassification of the last preoperative imaging according to 2018 LI-RADS protocol, that therefore did not influence clinical decision making: in this setting, HCC diagnosis followed the noninvasive EASL criteria [3].

Impaired hepatic functional reserve (Child Pugh score C, intractable ascites, platelet count < 50.000/µL) and high operative risk (American Association of Anesthesiologists score > 3) were considered as contraindications to SR.

Liver resection was performed either open or laparoscopically, according to patients and tumor characteristics. Regardless of the chosen approach, all patients underwent intraoperative ultrasound for tumor and vascular mapping. Intermittent pedicle clamping was selectively used, with no longer than 15 min of clamping time and at least 5 min of release.

Parenchymal transection was performed using the CUSA^®^ Excel + Cavitron Ultrasonic Surgical Aspirator System (Integra, Ireland) and the Ultracision Harmonic scalpel (Ethicon Endo-Surgery, Cincinnati, OH, USA), while hemostasis and biliostasis on the liver cut surface were achieved using metallic clips, Hem-o-lock or non-absorbable sutures.

HCC diagnosis was confirmed by pathological evaluation of the resected specimens in all patients, that following SR underwent life-long surveillance for HCC recurrence following the EASL guidelines [3]. LT represented a leading treatment option in patients who experienced a transplantable recurrence [21].

### 2.4. Study Endpoints

The primary endpoint of our study was to investigate the correlations between clinico-pathological characteristics of HCC patients and LI-RADS classes. As a secondary endpoint, we sought to analyze the risk factors affecting the oncological outcomes of SR.

### 2.5. Statistical Analysis

Continuous variables were described using median and interquartile range (IQR) while absolute numbers and proportions were reported for categorical variables. Baseline characteristics of LR-3/4 and LR-5 groups were compared using Mann–Whitney test and Chi-square test for continuous and categorical variables, respectively.

Uni- and multivariable Cox regression models were fitted for the time-to-event endpoints such as recurrence-free (RFS—time from resection to recurrence or death without recurrence) and overall survival (OS—time from resection to death), providing the hazard ratio (HR) and 95% confidence interval (95% CI) for identified risk factors.

Kaplan–Meier curves with the log-rank-test were also used to compare RFS and OS between LR-3/4 vs. LR-5 groups.

For the time to cancer-specific death, a competing regression analysis was performed considering death due to other causes as a competing event. The association of prognostic factors with this end-point was evaluated using cause-specific Cox models while Aalen–Johansen crude incidence curves with Gray test were estimated to compare LR-3/4 vs. LR-5 groups.

## 3. Results

**Figure 1:**
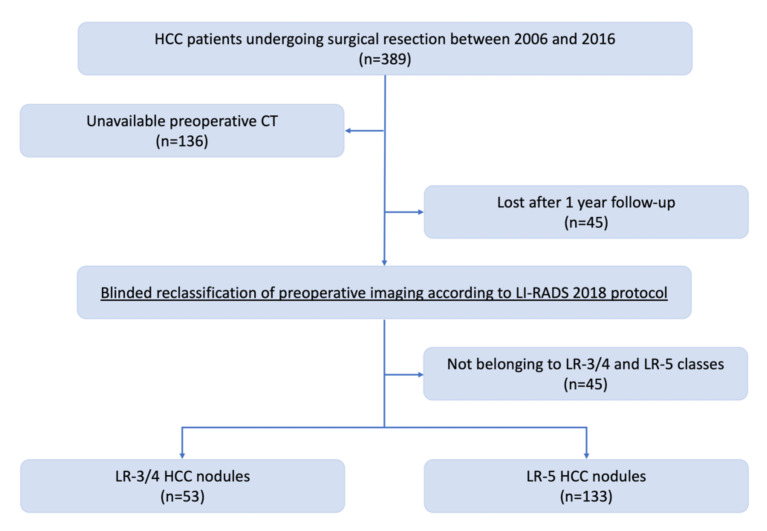
Flowchart for patient selection.

The flowchart for patient selection is depicted in Figure 1.

The starting population was composed of 389 HCC patients treated by SR between 2006 and 2016. Preoperative imaging was not retrievable for 136 patients that were therefore excluded from the analysis, while other 22 patients were excluded as classified as LR-TR-V, LR-TR-NV, LR-TR-E, LR-M and LR-TIV. Finally, we further excluded 45 patients that were lost after the first year of follow-up.

Our final population was composed of 186 patients, classified as LR-3/4 (53 patients) and 133 LR-5 (133 patients).

### 3.1. Patients and Tumor Characteristics

Clinical and demographic data of the whole study population are summarized in Table 1.

The median patient age was 66.6 (IQR: 58.3; 73.5) years, with 150 (80.6%) males and 36 (19.4%) females; 22 (11.8%) patients were classified as ECOG-PS 1.

One-hundred-three (55.4%) patients presented an HCV-related liver cirrhosis, 39 (21.0%) were HBV-positive while 35 (18.8%) were affected by alcoholic liver cirrhosis, while 9 (4.8%) patients shared other aetiologies (nonalcoholic-fatty-liver-disease, haemochromatosis, autoimmune or cryptogenic cirrhosis etc.).

Median MELD score of the entire cohort was 8.0 (IQR: 7.0; 10.0) points, with a median ALBI score of −2.5 (IQR: −2.80; −2.18) points, while 22 (13.1%) patients were classified as Child-Pugh B.

Median tumor size was 35.0 (IQR: 24.0; 51.5) mm, with 49 (26.3%) patients presenting multinodular disease; median log10αFP value was 1.17 (IQR: 0.70; 0.98) ng/mL.

One-hundred-eighteen (63.5%) patients were classified as BCLC-0/A, 57 (30.6%) patients belonged to BCLC-B class, while 11 (5.9%) were categorized as BCLC-C.

Fifty-three (28.5%) patients presented a LR-3/4 nodule, while the remaining 133 had a LR-5 HCC.

Seventy (38.3%) patients had a G3 tumor according to Edmondson–Steiner’s classification, MVI were detected in 67 (36.2%) specimens, with 39 (21.1%) patients presenting satellitosis and 43 (23.2%) capsular infiltration. A R0 resection was achieved in 153 (86.9%) patients.

Median hospital-stay lasted 9.0 (IQR: 7.0; 15.0) days, with a postoperative mortality of 2.2% and a 16.8% incidence of clinically relevant complications according to Clavien–Dindo classification.

One-hundred-twelve (60.2%) patients developed a tumor recurrence during follow-up, with 23 (12.5%) patients undergoing LT.

A total of 109 (58.6%) patients died, recording 55 (50.5%) tumor-related deaths.

### 3.2. Comparison of LR-3/4 and LR-5 Populations

Comparison of clinical and demographic data between LR-3/4 and LR-5 populations is summarized in Table 2.

LR-3/4 and LR-5 patients were homogeneous in terms of age (65.1 vs. 66.8 years; *p* = 0.941), ECOG-PS (13.2% vs. 11.3% ECOG-PS 1; *p* = 0.907), Child-Pugh class (14.9% vs. 12.4% Child-Pugh B; *p* = 0.86), MELD (8 vs. 8 points; *p* = 0.357) and ALBI (−2.44 vs. −2.51; *p* = 0.71) scores.

Similarly, the number (18.9% vs. 29.3% multinodular HCC; *p* = 0.202) and size (30 vs. 35 mm; *p* = 0.428) of nodules were comparable between the two subgroups.

Although tumor grading did not significantly differ between LR-3/4 and LR-5 nodules, LR-5 patients presented higher prevalence of MVI (22.6% vs. 41.7%; *p* = 0.024), satellitosis (9.4% vs. 25.8%; *p* = 0.024) and capsule infiltration (11.3% vs. 28%; *p* = 0.025).

The rate of R0 resections (94.0% vs. 84.1%; *p* = 0.132) and LT (13.2% vs. 12.2%; *p* = 1.000) did not differ between LR-3/4 and LR-5 populations.

### 3.3. Survival Analyses

Uni- and multivariate analyses of risk factors for RFS, OS and cancer-specific survival are reported in Table 3, Table 4 and Table 5.

Log10αFP (HR: 1.447; 95% CI: 1.138–1.840; *p* = 0.003), MVI (HR: 1.702; 95% CI: 1.079–2.686; *p* = 0.022), satellitosis (HR: 2.385; 95% CI: 1.382–4.118; *p* = 0.002), and R1 resection (HR: 2.891; 95% CI: 1.620–5.159; *p* < 0.001) resulted as independent risk factors for RFS on multivariate Cox regression analysis, while LI-RADS class did not exert any significant impact on such oncological outcome.

Focusing on OS, we identified patient age (HR: 1.038; 95% CI: 1.013–1.064; *p* = 0.003), ECOG-PS (HR: 2.457; 95% CI: 1.305–4.624; p0.005), MELD score (HR: 1.117; 95% CI: 1.007–1.239; *p* = 0.036), log10αFP (HR: 1.367; 95% CI: 1.024–1.825; 0.034), MVI (HR: 2.130; 95% CI: 1.281–3.540; *p* = 0.004), satellitosis (HR: 1.835; 95% CI: 1.064–3.116; *p* = 0.029) and R1 resection (HR: 3.540; 95% CI: 1.866–6.718; *p* < 0.001) as independent risk factors for overall survival on multivariate Cox regression analysis, without any impact of LI-RADS classification.

Last, MELD score (HR: 1.192; 95% CI: 1.037–1.369; *p* = 0.013), log10αFP (HR: 1.707; 95% CI: 1.138–2.559; *p* = 0.010), satellitosis (HR: 2.388; 95% CI: 1.188–4.802; *p* = 0.015) and R1 resection (HR: 3.463; 95% CI: 1.453–8.252; *p* = 0.005) resulted as independent risk factors for cancer-specific survival on multivariate competing regression analysis, while LI-RADS class did not exert any significant impact.

Kaplan–Meier estimates for RFS and OS and the Aalen–Johansen crude incidence estimates of cancer-specific death in the general population are shown in Supplementary Figures S1–S3.

**Figure 2:**
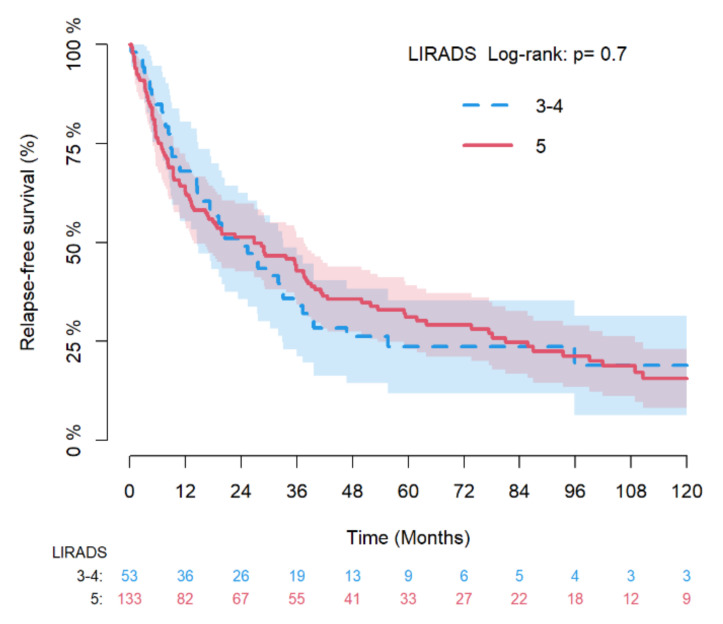
Kaplan–Meier estimates of relapse-free survival by LR-3/4 vs LR-5 with log-rank test.

**Figure 3:**
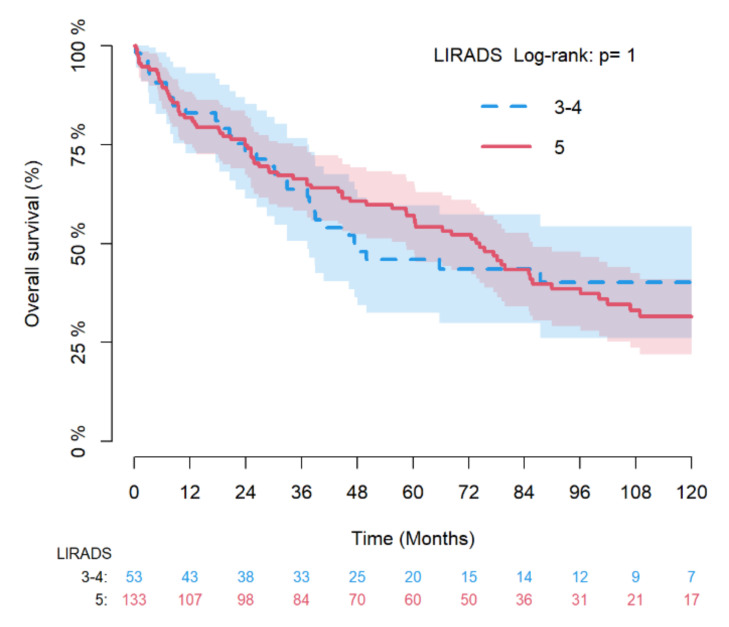
Kaplan–Meier estimates of overall survival by LR-3/4 vs LR-5 with log-rank test.

**Figure 4:**
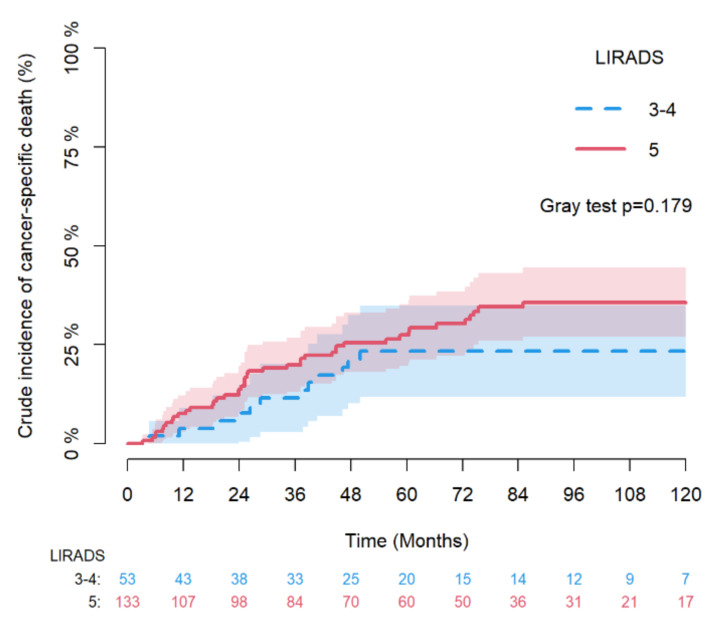
Aalen–Johansen crude incidence estimates of cancer-specific death with Gray test (death for other causes as competing risk) by LR-3/4 vs LR-5.

Figure 2 and Figure 3 depicts the Kaplan–Meier estimates for RFS and OS of LR-3/4 vs. LR-5 nodules with log-rank test and, while Figure 4 provides the Aalen–Johansen crude incidence estimates of cancer-specific death with Gray test (death for other causes as competing risk) stratified by LR-3/4 vs. LR-5.

## 4. Discussion

Contrast-enhanced imaging represents the milestone for HCC diagnosis.

According to current EASL guidelines, a nodule > 1 cm arising in a cirrhotic liver and presenting typical radiological hallmarks is eventually diagnosed as HCC. Such a binary approach has been challenged by the introduction of LI-RADS algorithm, which provides a probabilistic perspective to liver nodule diagnosis in at-risk patients, stratifying the probability of HCC (and overall malignancy) into specific classes.

Although the recent inclusion of LI-RADS protocol into AASLD guidelines represented a milestone towards the standardization of HCC diagnosis, there still is some concerns about the management of intermediate LI-RADS observations such as LR-3 (that potentially harbor an HCC focus in 38% of the cases) and LR-4 (with a 74% probability of HCC) nodules [6,22].

The prognostic value of LR-3 and LR-4 classes has been recently investigated in LT setting [9] through the blind application of LI-RADS categorization on a retrospective cohort of 245 transplanted HCC, highlighting a relatively high (40.9%) prevalence of such observations among all vital nodules detectable at last pre-LT imaging. Notably, inclusion of LR-3 and LR-4 categories (that should not be considered as HCC according to OPTN/UNOS policy [23,24]) into Metroticket 2.0 calculator [25] provided a higher prognostic accuracy, supporting the importance of such LI-RADS classes in the oncological outcomes of LT for HCC.

Similar conclusions have been suggested by another single-institution experience on a retrospective cohort of 72 patients undergoing LT [10], which highlighted a non-negligible prevalence of HCC (89%) and MVI (11%) in LR-4 observations, with not-statistically significant difference between LR-4 and LR-5 classes.

The oncological impact of LI-RADS classification has also been explored in the setting of PA and SR for single HCC [8]. After a blinded stratification of a highly selected (uninodular and treatment-naïve) retrospective cohort of 140 patients according to LI-RADS protocol, the Authors highlighted a significantly better outcome of SR over PA in LR-5 class, which was not observed in LR-3/4 subgroup of patients. These results were explained by a higher frequency of unfavorable pathological features in LR-5 classes, although not statistically significant.

The current study enrolled a relatively large cohort of 186 pathologically confirmed HCC treated by SR.

The first step of our analysis showed a higher prevalence of unfavorable histological findings in LR-5 class compared to LR-3/4 group, confirming a more aggressive pathological profiling of such class of observation and supporting the hypothesis that was raised in the above-mentioned study [8]. Moreover, it is important to highlight out how up to 22.6% of LR-3/4 observations in our cohort presented MVI, that has been already demonstrated as one of the most unfavorable histological features affecting worse oncological outcomes [26]. Another interesting finding was also represented by a non-negligible percentage of satellitosis (9.4%) and capsule infiltration (11.3%) even in LR-3/4 classes, supporting the need of cautious management of these intermediate-risk classes, as already suggested from other recently published studies [8,9,10,22].

The second step of our analysis sought to investigate the prognostic impact of several clinicopathological risk factors as well as the potential influence of LI-RADS classification on oncological outcomes of SR for HCC.

Our analysis confirmed the pivotal role of MVI [26], satellitosis [27], log10αFP [28] and R1 resection [29] on RFS, but failed to demonstrate an independent role of LI-RADS classification on this oncological outcome.

Interestingly, OS was significantly affected by impaired performance status and underlying liver function (as supported by the negative effect of higher MELD score and ECOG-PS 1) as well as being influenced by oncological characteristics such as the presence of MVI, satellitosis, higher log10αFP and R1 resection. Similarly to RFS, LI-RADS classification did not exert any statistically significant effect on OS.

Focusing on cancer-specific survival, we confirmed the detrimental effect of higher MELD score [30] as well as higher log10αFP, satellitosis and R1 resection on cancer-specific survival; once again, we did not observe any statistically significant association between LI-RADS class and cancer-specific outcomes.

In summary, our results confirmed the current evidence concerning the risk factors for cancer-specific outcomes after curative resection for HCC [31].

Despite the observed association of LR-5 class with several unfavorable pathological features, the absence of an independent correlation between LI-RADS classification and oncological results of SR could rely on the higher relative weight of tumor pathology and underlying tumorigenic liver asset in the determination of oncological results.

The main limitations of this study are represented by its retrospective nature, which may imply selection and indication biases, as well as the loss of many cases because of irretrievable preoperative imaging (196/349 patients; 56.16%) that caused a significant decrease in the study population.

## 5. Conclusions

In conclusion, the current study supported the hypothesis of a significant association of higher LI-RADS class with unfavorable pathological features such as MVI, satellitosis or capsule infiltration [8,32], confirming the detrimental effect of tumor pathology and margin-status on cancer-specific outcomes after SR for HCC [26,27,28,29,30,31,33].

Another interesting finding was the identification of a non-negligible percentage of MVI (22.6%), satellitosis (9.4%) and capsule infiltration (11.3%) in LR-3/4 observations, supporting the need for a cautious evaluation and management of such intermediate-risk classes, as already advocated by other authors [8,9,10,22].

Our results reinforce the role of preoperative radiological assessment as a predictor of tumor biology, a field that will find further application with the development and implementation of radiomics in the upcoming precision-medicine era [34,35].

## Figures and Tables

**Table 1:** Overall description of patients and tumor characteristics.

Variable	Overall N = 186	Missings (%)
Age at surgery, years (median [IQR])	66.63 [58.30; 73.50]	0 (0)
Sex = Female (%)	36 (19.4)	0 (0)
Performance Status = 1 (%)	22 (11.8)	0 (0)
Alcoholic liver cirrhosis (%)	35 (18.8)	0 (0)
HBV-related liver cirrhosis (%)	39 (21.0)	0 (0)
HCV-related liver cirrhosis (%)	103 (55.4)	0 (0)
Child Pugh class B (%)	22 (13.8)	18 (9.7%)
MELD score, points (median [IQR])	8.00 [7.00; 10.00]	0 (0)
ALBI score, points (median [IQR])	−2.50 [−2.80; −2.18]	1 (0.5)
ALBI grade 1 (%)	76 (41.1)	1 (0.5)
ALBI grade 2 (%)	102 (55.1)	1 (0.5)
ALBI grade 3 (%)	7 (3.8)	1 (0.5)
log10αFP, ng/mL (median [IQR])	1.17 [0.70; 0.98]	25 (13.4)
Multinodular tumor (%)	49 (26.3)	0 (0)
Maximum tumor size, mm (median [IQR])	35.00 [24.00; 51.50]	0 (0)
Satellitosis (%)	37 (19.9)	0 (0)
BCLC = 0 (%)	28 (15.1)	0 (0)
BCLC = A (%)	90 (48.4)	0 (0)
BCLC = B (%)	57 (30.6)	0 (0)
BCLC = C (%)	11 (5.9)	0 (0)
LI-RADS = 3 (%)	17 (9.1)	0 (0)
LI-RADS = 4 (%)	36 (19.4)	0 (0)
LI-RADS = 5 (%)	133 (71.5)	0 (0)
Clavien–Dindo Score = 0 (%)	106 (57.6)	2 (1.1)
Clavien–Dindo Score = 1 (%)	16 (8.7)	2 (1.1)
Clavien–Dindo Score = 2 (%)	30 (16.3)	2 (1.1)
Clavien–Dindo Score = 3a (%)	7 (3.8)	2 (1.1)
Clavien–Dindo Score = 3b (%)	3 (1.6)	2 (1.1)
Clavien–Dindo Score = 4a (%)	10 (5.4)	2 (1.1)
Clavien–Dindo Score = 4b (%)	7 (3.8)	2 (1.1)
Clavien–Dindo Score = 5 (%)	4 (2.2)	2 (1.1)
Length of stay (median [IQR])	9.00 [7.00; 15.00]	0 (0)
Tumor grading = 1 (%)	17 (9.3)	3 (1.6)
Tumor grading = 2 (%)	96 (52.5)	3 (1.6)
Tumor grading = 3 (%)	70 (38.3)	3 (1.6)
Microvascular invasion (%)	76 (36.2)	1 (1.1)
Satellitosis (%)	39 (21.1)	1 (0.5)
Capsule infiltration (%)	43 (23.2)	1 (0.5)
R0 resection (%)	153 (86.9)	10 (5.4)
Tumor recurrence (%)	112 (60.2)	0 (0)
Liver transplant after resection (%)	23 (12.5)	2 (1.1)
Patient death (%)	109 (58.6)	0 (0)
Cause of Patient death = Cirrhosis (%)	29 (26.6)	0 (0)
Cause of Patient death = Tumor (%)	55 (50.5)	0 (0)
Cause of Patient death = Other (%)	25 (22.9)	0 (0)

Abbreviations: MELD, Model for End Stage Liver Disease; HBV, Hepatitis B Virus; HCV, Hepatitis C Virus; ALBI, Albumin-Bilirubin; αFP, alpha-fetoprotein; BCLC, Barcelona Clinic for Liver Cancer; LI-RADS, Liver Imaging Reporting and Data System.

**Table 2:** Comparison of patients and tumor characteristics by LR-3/4 vs LR-5.

Variable	LR-3/4 N = 53	LR-5 N = 133	*p*-Value ^1^
Age, years (median [IQR])	65.15 [58.69; 73.39]	66.81 [57.72; 73.69]	0.941
Sex = Female (%)	10 (18.9)	26 (19.5)	1.000
Performance Status = 1 (%)	7 (13.2)	15 (11.3)	0.907
Child Pugh class B (%)	7 (14.9)	15 (12.4)	0.86
MELD score, points (median [IQR])	8.00 [7.00; 10.00]	8.00 [7.00; 10.00]	0.357
ALBI score, points (median [IQR])	−2.44 [−2.87; −2.09]	−2.51 [−2.77; −2.27]	0.714
ALBI grade (%) ^2^	-	-	0.693
ALBI grade 1 (%)	21 (39.6)	55 (41.7)	-
ALBI grade 2 (%)	29 (54.7)	73 (55.3)	-
ALBI grade 3 (%)	3 (5.7)	4 (3.0)	-
log10αFP, ng/mL (median [IQR])	0.95 [0.63; 1.35]	1.30 [0.75; 2.09]	0.028
Multinodular tumor (%)	10 (18.9)	39 (29.3)	0.202
Maximum tumor size, mm (median [IQR])	30.00 [23.00; 50.00]	35.00 [24.00; 55.00]	0.428
BCLC (%) ^3^	-	-	0.322
BCLC = 0 (%)	11 (20.8)	17 (12.8)	-
BCLC = A (%)	26 (49.1)	64 (48.1)	-
BCLC = B (%)	12 (22.6)	45 (33.8)	-
BCLC = C (%)	4 (7.5)	7 (5.3)	-
Tumor grading (%) ^4^	-	-	0.178
Tumor grading = 1 (%)	8 (15.4)	9 (6.9)	-
Tumor grading = 2 (%)	24 (46.2)	72 (55.0)	-
Tumor grading = 3 (%)	20 (38.5)	50 (38.2)	-
Microvascular invasion (%)	12 (22.6)	55 (41.7)	0.024
Satellitosis (%)	5 (9.4)	34 (25.8)	0.024
Capsule infiltration (%)	6 (11.3)	37 (28.0)	0.025
R0 resection (%)	47 (94.0)	106 (84.1)	0.132
Liver transplant after resection (%)	7 (13.2)	16 (12.2)	1.000

^1^ Chi-square or Mann–Whitney test *p*-value; ^2^ *p*-value is referred to ALBI grade 1,2,3 in the two groups; ^3^ *p*-value is referred to BCLC 0, A, B, C in the two groups; ^4^ *p*-value is referred to tumor grading 1, 2, 3 in the two groups. Abbreviations: MELD, Model for End Stage Liver Disease; ALBI, Albumin-Bilirubin; αFP, alpha-fetoprotein; BCLC, Barcelona Clinic for Liver Cancer.

**Table 3:** Univariate and multivariate association of risk factors with relapse-free survival by Cox regression.

Variable	Univariate Model		Multivariate Model	
	HR (95% CI)	*p*-Value	HR (95% CI)	*p*-Value
Age, per 10 years	1.154 (0.987–1.349)	0.073	1.020 (0.999–1.041)	0.061
Sex: female vs. male	1.066 (0.714–1.594)	0.754	-	-
Performance status: 1 vs. 0	2.28 (1.426–3.644)	0.001	1.766 (0.982–3.177)	0.057
Child Pugh class: B vs. A	1.81 (1.119–2.928)	0.016	1.276 (0.640–2.541)	0.489
MELD score, per unit	1.047 (0.982–1.117)	0.161	-	-
ALBI score, per unit	1.555 (1.125–2.150)	0.007	1.346 (0.821–2.205)	0.239
log10αFP, per unit	1.372 (1.159–1.625)	<0.001	1.447 (1.138–1.840)	0.003
Multinodular tumor vs. single tumor	2.093 (1.459–3.002)	<0.001	1.424 (0.841–2.409)	0.188
Maximum nodule size, per unit	1.000 (0.994–1.006)	0.963	-	-
BCLC B–C vs. 0–A	1.155 (0.823–1.619)	0.405	-	-
Tumor grading 3 vs. 1–2	2.023 (1.451–2.821)	<0.001	1.286 (0.802–2.064)	0.297
Microvascular invasion yes vs. no	2.214 (1.581–3.099)	<0.001	1.702 (1.079–2.686)	0.022
Satellitosis yes vs. no	2.884 (1.935–4.298)	<0.001	2.385 (1.382–4.118)	0.002
Capsule infiltration yes vs. no	1.521 (1.050–2.203)	0.027	0.967 (0.580–1.611)	0.897
R1 resection vs. R0 resection	3.178 (2.004–5.041)	<0.001	2.891 (1.620–5.159)	<0.001
LI-RADS 5 vs LI-RADS 3–4	0.939 (0.655–1.345)	0.730	0.769 (0.485–1.218)	0.263

Abbreviations: MELD, Model for End Stage Liver Disease; ALBI, Albumin-Bilirubin; αFP, alpha-fetoprotein; BCLC, Barcelona Clinic for Liver Cancer; LI-RADS, Liver Imaging Reporting and Data System.

**Table 4:** Uni- and multivariate association of risk factors with overall survival by Cox regression.

Variable	Univariate Model		Multivariate Model	
	HR (95% CI)	*p*-Value	HR (95% CI)	*p*-Value
Age, per 10 years	1.281 (1.060–1.549)	0.010	1.038 (1.013–1.064)	0.003
Sex: female vs. male	0.997 (0.624–1.592)	0.990	-	-
Performance status: 1 vs. 0	2.679 (1.624–4.419)	<0.001	2.457 (1.305–4.624)	0.005
Child Pug: B vs. A	2.284 (1.362–3.833)	0.002	2.398 (1.128–5.097)	0.023
MELD score, per unit	1.090 (1.016–1.169)	0.016	1.117 (1.007–1.239)	0.036
ALBI score, per unit	1.573 (1.077–2.296)	0.019	0.808 (0.452–1.444)	0.471
log10αFP, per unit	1.343 (1.101–1.638)	0.004	1.367 (1.024–1.825)	0.034
Multinodular tumor vs. single tumor	1.729 (1.146–2.608)	0.009	1.253 (0.697–2.250)	0.451
Maximum nodule size, per unit	1.002 (0.997–1.008)	0.410	-	-
BCLC B–C vs. 0–A	1.329 (0.904–1.952)	0.148	-	-
Tumor grading 3 vs. 1–2	2.151 (1.471–3.146)	<0.001	1.265 (0.748–2.141)	0.381
Microvascular invasion yes vs. no	2.388 (1.633–3.491)	<0.001	2.130 (1.281–3.540)	0.004
Satellitosis yes vs. no	2.690 (1.758–4.144)	<0.001	1.835 (1.064–3.166)	0.029
Capsule infiltration yes vs. no	1.396 (0.916–2.128)	0.121	-	-
R1 resection vs. R0 resection	2.636 (1.574–4.414)	<0.001	3.540 (1.866–6.718)	<0.001
LI-RADS 5 vs LI-RADS 3–4	1.009 (0.662–1.536)	0.968	0.862 (0.493–1.506)	0.602

Abbreviations: MELD, Model for End Stage Liver Disease; ALBI, Albumin-Bilirubin; αFP, alpha-fetoprotein; BCLC, Barcelona Clinic for Liver Cancer; LI-RADS, Liver Imaging Reporting and Data System.

**Table 5:** Univariate and multivariate association of risk factors with cancer-specific death by Cox cause-specific regression considering death by other causes as a competing risk.

Variable	Univariate Model		Multivariate Model	
	HR (95% CI)	*p*-Value	HR (95% CI)	*p*-Value
Age, per 10 years	1.074 (0.842–1.370)	0.564	1.032 (0.997–1.068)	0.071
Sex: female vs. male	0.879 (0.443–1.745)	0.713	-	-
Performance status: 1 vs. 0	2.431 (1.186–4.981)	0.015	1.803 (0.728–4.464)	0.203
Child Pug: B vs. A	3.054 (1.589–5.867)	0.001	2.072 (0.733–5.860)	0.170
MELD score, per unit	1.160 (1.061–1.269)	0.001	1.192 (1.037–1.369)	0.013
ALBI score, per unit	2.262 (1.350–3.791)	0.002	1.005 (0.437–2.313)	0.991
log10αFP, per unit	1.597 (1.235–2.065)	<0.001	1.707 (1.138–2.559)	0.010
Multinodular tumor vs. single tumor	2.261 (1.302–3.925)	0.004	1.144 (0.521–2.514)	0.737
Maximum nodule size, per unit	1.003 (0.995–1.011)	0.442	-	-
BCLC B–C vs. 0–A	1.508 (0.882–2.577)	0.133	-	-
Tumor grading 3 vs. 1–2	2.853 (1.671–4.871)	<0.001	1.844 (0.870–3.907)	0.110
Microvascular invasion yes vs. no	2.901 (1.703–4.941)	<0.001	1.948 (0.938–4.049)	0.074
Satellitosis yes vs. no	4.097 (2.365–7.098)	<0.001	2.388 (1.188–4.802)	0.015
Capsule infiltration yes vs. no	1.073 (0.576–2.000)	0.823	-	-
R1 resection vs. R0 resection	3.477 (1.775–6.811)	<0.001	3.463 (1.453–8.252)	0.005
LI-RADS 5 vs LI-RADS 3–4	1.361 (0.718–2.582)	0.345	0.829 (0.383–1.795)	0.634

Abbreviations: MELD, Model for End Stage Liver Disease; ALBI, Albumin-Bilirubin; αFP, alpha-fetoprotein; BCLC, Barcelona Clinic for Liver Cancer; LI-RADS, Liver Imaging Reporting and Data System.

## Data Availability

Data will be available upon request to the corresponding author.

## Supplementary Materials

The following are available online at https://www.mdpi.com/article/10.3390/diagnostics12010160/s1, Figure S1: Kaplan–Meier estimates of relapse-free survival, Figure S2: Kaplan–Meier estimates of overall survival, Figure S3: Aalen–Johansen crude incidence estimates of cancer-specific death (death for other causes as competing risk).
