# Supplementary material for: From LI-RADS Classification to HCC Pathology: A Retrospective Single-Institution Analysis of Clinico-Pathological Features Affecting Oncological Outcomes after Curative Surgery
Source: Diagnostics (Basel). 2022 Jan 10;12(1):160. doi: 10.3390/diagnostics12010160 (PMC8775107; doi:10.3390/diagnostics12010160)
Supplement: Supplementary file 1 [file diagnostics-12-00160-s001.zip › diagnostics-1512301-supplementary.pdf]

Figure S1. Kaplan-Meier estimates of relapse-free survival.

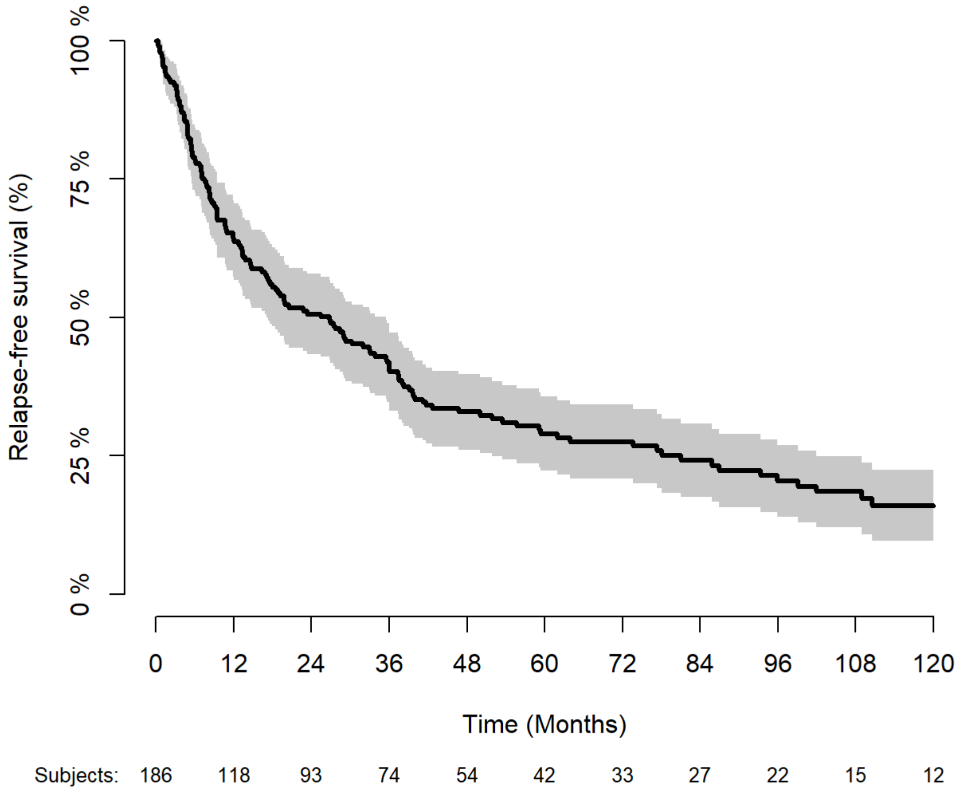

	time	n.risk	n.event	n.lost	surv	se.surv	lower	upper
1	12	118	0	0	0.642	0.0353	0.5729	0.711
2	24	93	0	0	0.506	0.0369	0.4339	0.578
3	36	74	0	0	0.407	0.0363	0.3363	0.478
4	48	54	0	0	0.329	0.0348	0.2608	0.397
5	60	42	0	0	0.289	0.0342	0.2225	0.357
6	120	12	0	0	0.160	0.0328	0.0961	0.224

Figure S2. Kaplan-Meier estimates of overall survival.

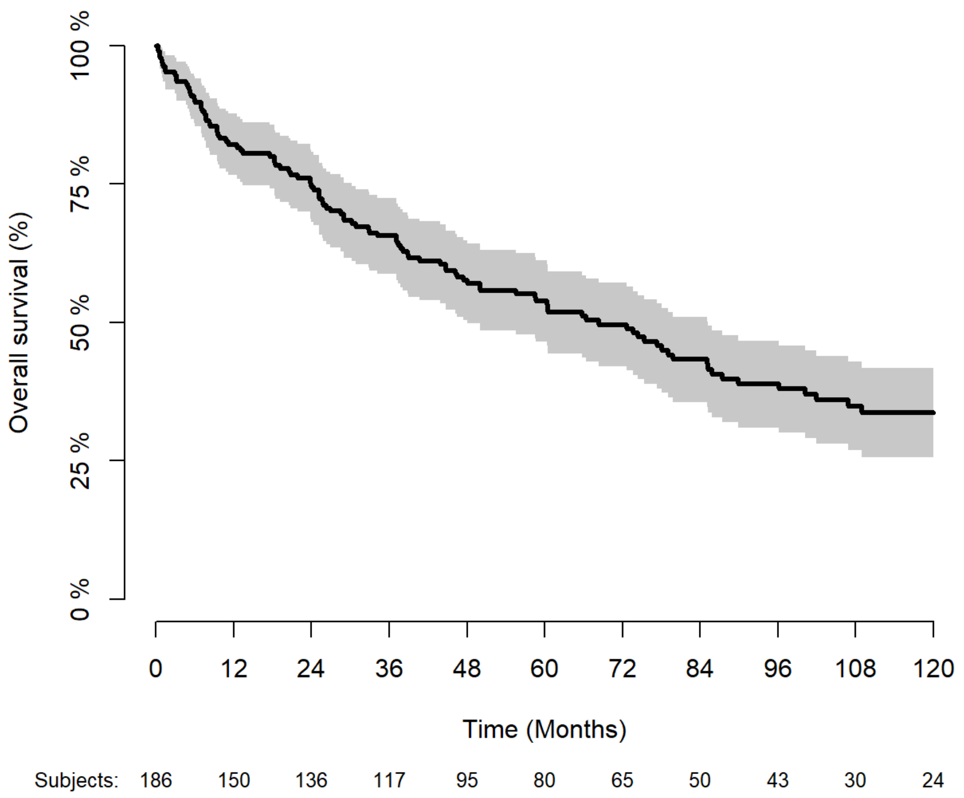

	time	n.risk	n.event	n.lost	surv	se.surv	lower	upper
1	12	150	0	0	0.821	0.0282	0.766	0.876
2	24	136	0	0	0.744	0.0322	0.681	0.808
3	36	117	0	0	0.656	0.0351	0.587	0.725
4	48	95	0	0	0.576	0.0368	0.504	0.648
5	60	80	0	0	0.538	0.0375	0.464	0.612
6	120	24	0	0	0.337	0.0410	0.257	0.417

Figure S3. Aalen-Johansen crude incidence estimates of cancer-specific death (death for other causes as competing risk).

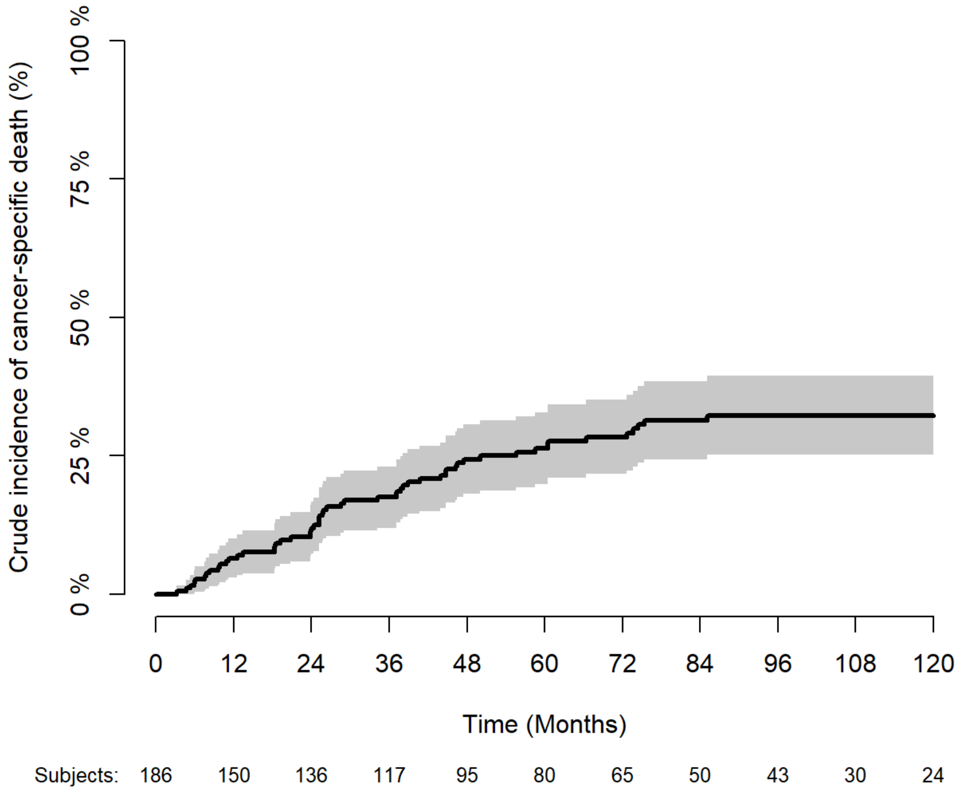

	time	n.risk	n.event	n.lost	cuminc	se.cuminc	lower	upper
1	12	150	0	0	0.0652	0.0182	0.0295	0.101
2	24	136	0	0	0.1199	0.0240	0.0729	0.167
3	36	117	0	0	0.1749	0.0281	0.1199	0.230
4	48	95	0	0	0.2437	0.0320	0.1810	0.306
5	60	80	0	0	0.2629	0.0331	0.1981	0.328
6	120	24	0	0	0.3230	0.0365	0.2514	0.395
